# Mortality, disability, and healthcare expenditure of patients with seropositive rheumatoid arthritis in Korea: A nationwide population-based study

**DOI:** 10.1371/journal.pone.0210471

**Published:** 2019-01-08

**Authors:** In Ah Choi, Jeong Seok Lee, Yeong Wook Song, Eun Young Lee

**Affiliations:** 1 Division of Rheumatology, Department of Internal Medicine, Chungbuk National University Hospital, Cheongju, Republic of Korea; 2 Division of Rheumatology, Department of Internal Medicine, Seoul National University College of Medicine, Seoul, Republic of Korea; 3 Graduate School of Medical Science and Engineering, Korea Advanced Institute of Science and Technology, Daejeon, Republic of Korea; University of Twente, NETHERLANDS

## Abstract

**Background:**

We investigated the mortality and disability rate, as well as the healthcare expenditure, for patients with newly diagnosed seropositive rheumatoid arthritis (RA) who were followed-up for up to 10 years, compared to the general population in Korea.

**Methods:**

We conducted a nationwide population-based study using a National Health Insurance Service-National Sample Cohort of the Korean population, consisting of 1 million individuals who submitted medical care claims between 2002 and 2013. RA was identified using as the International Classification of Diseases code M05 (seropositive RA), with prescription of any disease-modifying anti-rheumatic drug (DMARD). Our analysis was based on the data of 1655 patients with incident seropositive RA and 8275 non-RA controls. The controls were matched to the RA cohort by sex, age at the time of diagnosis, duration of follow-up, geographic region, type of social security, and household income.

**Results:**

The most commonly used conventional synthetic DMARDs were hydroxychloroquine (71.30%) and methotrexate (69.5%), with adalimumab being the most commonly used biologic DMARD (2.54%). The mortality rate was significantly higher in the RA than the control group (incidence rate ratio [IRR] 1.29, 95% confidence interval [CI] 1.02–1.64) in the first 10 years after diagnosis. Specifically, mortality due to infectious diseases (IRR 4.41, 95% CI 1.60–12.17) and pneumonia (IRR 3.92, 95% CI 1.46–10.53) was significantly higher in the RA than control group. The disability rate was higher in the RA than control group over the first 10 years of the disease (IRR 2.27, 95% CI 1.77–2.92), which was attributed to a higher incidence of physical disability (IRR 3.81, 95% CI 2.81–5.15). Annual health expenditure was greater for the RA than the control group.

**Conclusions:**

Therefore, the rate of mortality and disability, as well as healthcare expenditure, are higher for patients with RA over the first 10 years of the disease onset, than the general population of Korea. The use of claim data has limited the quality of information and there is a limit to the observation period, and we expect the prospective national-wide multicenter cohort for longer period to overcome these limitations.

## Introduction

Rheumatoid arthritis (RA) is a chronic inflammatory disease that leads to functional disability [1] and premature mortality [2–4]. Although the long-term prognosis of RA has improved dramatically following the introduction of effective disease modifying anti-rheumatic drugs (DMARDs), including biologic agents and treat-to-target approach for remission, the overall global burden of RA is increasing, owing to its chronic progressive course and the increasing life expectancy worldwide [5]. This burden is largely contributed by RA-associated mortality and disability, but also includes the economic burden of RA treatment and management of its comorbidities.

Since medical insurance coverage and social security systems vary widely between countries, the burden of RA is expected to vary greatly from country to country. In this study, we aimed to identify the status of disability and mortality in patients with newly diagnosed seropositive RA, who were followed-up for up to 10 years, as compared to the general population in Korea.

## Methods

### Study design

This was a nationwide population-based, nested case-control study of patients with RA and matched controls. Mortality, disability, and healthcare costs were investigated as primary outcomes. Reporting of this manuscripts adheres to the REporting of studies Conducted using Observational Routinely-collected Data (RECORD) checklist and the completed checklist are available in the supplementary text (S1 Text).

### Data sources

Our study was based on the National Health Insurance Service-National Sample Cohort (NHIS-NSC), which is a Korean population-based cohort of individuals who submitted medical care claims, between 2002 and 2013 [6]. The cohort, which consisted of 1,025,340 individuals, was obtained through age-, sex- and income-based stratification sampling of approximately 50 million Koreans who were registered with the Korean NHIS in 2002. Because the NHIS-NSC data set contains only de-identified secondary data released for research purposes, the present study was exempt from ethical review by the institutional review board of Chungbuk National University Hospital (CBNUH IRB No. 2018-06-015) and Seoul National University Hospital (SNUH IRB No. 1806-075-951). For the same reason, patient consent was not required for the present analysis. All individuals included in the NSC provided informed consent for data collection, storage, analysis, and publication.

### Study variables

A diagnosis of RA was defined by the diagnostic code for seropositive RA (M05), with the prescription of any disease-modifying anti-rheumatic drug (DMARD), as per previously reported methodology [7]. The controls were matched to the RA cohort by sex, age at the time of diagnosis, duration of follow-up, geographic region, type of social security, and household income.

Mortality, disability, and annual healthcare costs were analyzed as dependent variables. With regard to disability, the only information from the Disability Registration System, operated by the Ministry of Health and Welfare in Korea that is available in the NHIS-NSC database is the presence or absence of disability and categories of disability, such as ‘physical disability’, but not the grades of disability. We evaluated the presence and extent of disability using the specific guidelines, provided by government, with confirmation from the treating physician. The specific criteria for physical disability due to joint disorders, including RA, have been provided in the supplementary text (S2 Text). The annual healthcare costs covered by the NHIS in Korea includes medical costs for diagnostic tests; treatment, including surgery, intervention, or medication; and outpatient or inpatient care. South Korea has a universal health coverage system, with approximately 98% of the Korean population registered in the NHIS, and over 99% of claims, since 2005, having been processed through the automated billing system [8]. Therefore, the annual healthcare costs entered into the system are considered to be almost equal to the direct medical cost. RA-related and -unrelated costs were evaluated separately, based on the presence of the diagnosis code for RA.

### Statistical analysis

The sociodemographic features of the RA and control groups are presented in terms of the number of patients and the prevalence of mortality and disability. Mortality and disability rates were calculated per 1000 person-years (PY) and used to calculate incidence rate ratios (IRR), with the associated 95% confidence intervals (CI), thus establishing the risk for a certain outcome in the study group compared to the corresponding risk in the control cohort. The median and interquartile range of annual health costs were calculated and compared between the study and the control cohort, using the Mann-Whitney U-test. The SAS Enterprise Guide (version 5.1; SAS Institute Inc., Cary, NC, USA) was used for the analysis.

The temporal trend in the health expenditure per capita (USD) was examined using the Joinpoint regression program [9]. This program uses the Bayesian information criterion to generate different numbers of joinpoints, indicating time points at which the rate or hazard of occurrence of an event changes significantly and to fit separate linear trends in each time segment. Annual percentage changes (APCs) for each segment were calculated.

## Results

### Selection of patients and controls

For the initial step of patient extraction, patients with diagnosis code of M05, according to the 10^th^ version of the International Classification of Diseases (ICD-10), were identified (n = 9907). After exclusion of those without a prescription for any DMARD, 2870 patients with RA were retained.

The first 2 years (2002–2003) were considered as a washout period, as we focused on analyzing the burden of incident RA. After applying this selection criteria, 2316 patients with incident RA (i.e., diagnosed between 2004 and 2013) were selected. After this exclusion, 658 patients who had a prior diagnosis of another rheumatic disease, such as systemic connective tissue disorders (ICD-10 code: M30-M36), ankylosing spondylitis (ICD-10 code: M45), psoriatic, and enteropathic arthropathies (ICD-10 code: M07), or juvenile arthritis (ICD-10 code: M08), as well as 3 patients who were diagnosed with RA before the age of 20 years, the final study group of 1655 patients was established and the data extracted from the NHIS-NSC for analysis.

**Fig 1:**
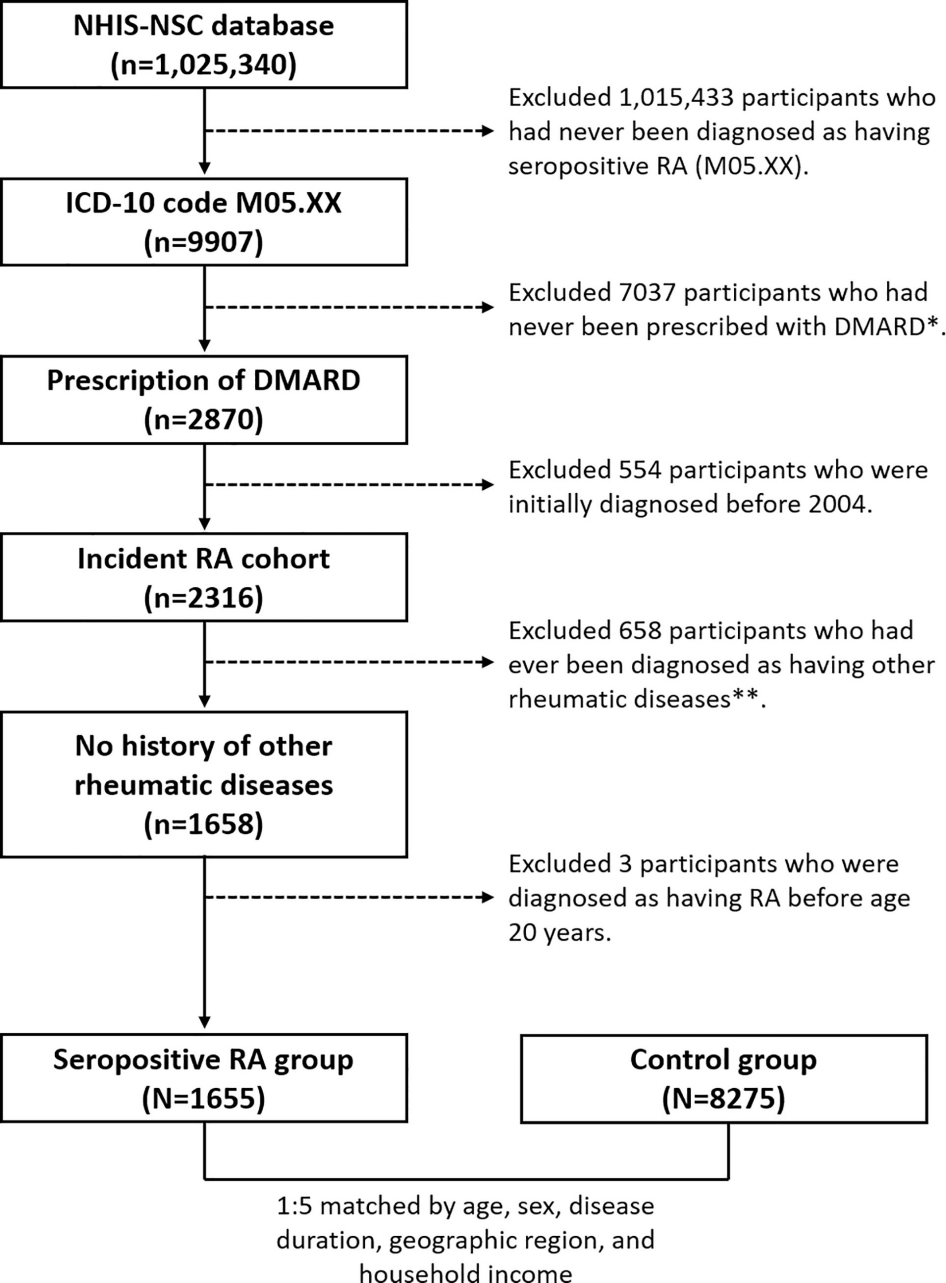
Flowchart of the selection of study participants. *Methotrexate, sulfasalazine, hydroxychloroquine, leflunomide, tacrolimus, bucillamine, mizoribine, cyclosporine, infliximab, etanercept, adalimumab, golimumab, abatacept, tocilizumab, rituximab, and tofacitinib. **Systemic connective tissue disorders (International Classification of Diseases 10th revision [ICD-10] code: M30-M36), ankylosing spondylitis (ICD-10 code: M45), psoriatic and enteropathic arthropathies (ICD-10 code: M07), or juvenile arthritis (ICD-10 code: M08). RA, rheumatoid arthritis; DMARD, disease-modifying anti-rheumatic drug.

Additionally, 8275 controls (1:5 matched by age, sex, and income status to each RA patient) were selected from among individuals insured from 2004 to 2013 (Fig 1).

### Sociodemographic and clinical characteristics of the RA and control groups at diagnosis

The sociodemographic characteristics of the RA group, at the time of diagnosis, and of the control group are summarized in Table 1. The distribution of sex, age, follow-up duration, geographic region, household income, and the type of social security, according to the household income, was identical in the RA and control group, indicative that appropriate matching was achieved. More patients in RA group used glucocorticoid more than 4 weeks compared to controls.

**Table 1:** Sociodemographic characteristics of the RA and control groups.

Characteristics	RA group (n = 1655)	Control group (n = 8275)
**Sex**		
**Male (% of group)**	378 (22.84%)	1890 (22.84%)
**Age, years (% of group)**	**At the time of diagnosis**	
**20–29**	34 (2.54%)	170 (2.54%)
**30–39**	162 (9.79%)	810 (9.79%)
**40–49**	335 (20.24%)	1675 (20.24%)
**50–59**	513 (41.00%)	2565 (41.00%)
**60–69**	383 (23.14%)	1915 (23.14%)
**70–79**	199 (12.02%)	995 (12.02%)
**80-**	29 (1.75%)	145 (1.75%)
**Duration of follow-up, years (% of group)**		
**<5**	959 (57.95%)	4795 (57.95%)
**≥5**	696 (42.05%)	3480 (42.05%)
**Geographic region**		
**Seoul, metropolitan**	299 (18.07%)	1495 (18.07%)
**Large cities**^**a**^	459 (27.73%)	2295 (27.73%)
**Other areas**	897 (54.20%)	4485 (54.20%)
**Type of social security**^**b**^		
**Health insurance**	1581 (95.53%)	7905 (95.53%)
**Medical aid**	74 (4.47%)	370 (4.47%)
**Household income**		
**1st quintile**	211(12.75%)	1055(12.75%)
**2nd quintile**	237 (14.32%)	1185 (14.32%)
**3rd quintile**	271 (16.37%)	1355 (16.37%)
**4th quintile**	387 (23.38%)	1935 (23.38%)
**5th quintile, highest**	475 (28.70%)	2375 (28.70%)
**Glucocorticoid use longer** **than 4 weeks**	1361 (82.24%)	1529 (18.48%)
**Prevalence of** **Comorbidities**		
**Diabetes mellitus**	636 (38.43%)	2522 (30.48%)
**Thyroid disease**	810 (48.94%)	2494 (30.14%)

^a^Including 6 major cities (Busan, Daegu, Incheon, Gwangju, Daejeon, and Ulsan) in Korea.

^b^The type of social security at the time of diagnosis was determined according to the household income level. RA, rheumatoid arthritis.

In the RA group, the most commonly used DMARD was hydroxychloroquine (71.30%), followed by methotrexate (69.49%), sulfasalazine (36.13%), leflunomide (26.22%), and bucillamine (16.25%). The most commonly used biologic DMARD was adalimumab (2.54%), followed by etanercept (1.75%), infliximab (0.85%), and rituximab (0.48%).

### Mortality and disability rates

A comparison of mortality rates between the RA and control group is shown in Table 2. Over the follow-up period, 86 patients with RA, in 6700 person-years, and 335 controls, in 33,787 person-years died, presenting significant higher mortality in RA in the first 10 years of the disease (IRR 1.29, 95% CI 1.02–1.64). The significantly higher mortality rate due to infections (IRR 4.41, 95% CI 1.60–12.17) and respiratory disease (IRR 2.10, 95% CI 1.00–4.39), especially pneumonia (IRR 3.92, 95% CI 1.46–10.53), as well as musculoskeletal disease (1.64 per 1000 person-years, cause of death in all 11 cases were RA) contributed to the higher mortality of RA.

**Table 2:** Comparison of mortality between the RA and control groups.

	RA (6700 PY)		Control (33,787 PY)		Incidence rate ratio
Cause of death (ICD-10 code)	Number of cases	Per 1000 person-years	Number of cases	Per 1000 person-years	95% confidence interval
All cause	86	12.84	335	9.92	1.29 (1.02–1.64)
Infection (A00-B99)	7	1.04	8	0.24	4.41 (1.60–12.17)
Neoplasm (C00-D48)	16	2.39	122	3.61	0.66 (0.39–1.11)
Endocrine (E00-E90)	2	0.30	14	0.41	0.72 (0.16–3.17)
Nervous (G00-G99)	3	0.45	10	0.30	1.51 (0.42–5.50)
Circulatory (I00-I99)	13	1.94	72	2.13	0.91 (0.50–1.64)
Respiratory (J00-J99)	10	1.49	24	0.71	2.10 (1.00–4.39)
Pneumonia (J12-J18)	7	1.04	9	0.27	3.92 (1.46–10.53)
Digestive (K00-K93)	3	0.45	11	0.33	1.38 (0.38–4.93)
Musculoskeletal (M00-M99)	11	1.64	0	0.00	-
Genitourinary (N00-N99)	3	0.45	9	0.27	1.68 (0.46–6.21)
Injury, poisoning and other external (S00-T88)	8	1.19	35	1.04	1.15 (0.53–2.48)
Others (R00-99 and not specified)	10	1.49	30	0.89	1.68 (0.82–3.44)

RA, rheumatoid arthritis; PY, person-year; ICD-10, International Classification of Diseases 10th Revision

Both in the RA and control groups, the main cause of death was malignancy (2.39 and 3.61 per 1000 person-years, respectively), followed by cardiovascular disease (1.94 and 2.13 per 1000 person-years, respectively), while there was no significant difference in the prevalence between RA and controls.

A comparison of the rate, type and distribution of disabilities between the RA and control group is provided in Table 3. The disability rate was higher in the RA than control group in the first 10 years of the disease (IRR 2.27, 95% CI 1.77–2.92), with this difference being mainly attributed to physical disability, which was significantly higher in RA than control group (IRR 3.81, 95% CI 2.81–5.15). Non-physical disability was not significantly different between the RA and control group (IRR 0.76, 95% CI 0.45–1.32).

**Table 3:** Comparison of rate, type, and distribution of disability between the RA and control groups.

	RA (6409 person-person-year)		Control (33,085 person-year)		Incidence rate ratio
	Case	Per 1000 PY	Case	Per 1000 Per Person Year	95% CI
**All disability**	88	13.73	200	6.05	2.27 (1.77–2.92)
**Physical disability**	73	11.39	99	2.99	3.81 (2.81–5.15)
**Non-physical disability**	15	2.32	101	3.05	0.76 (0.45–1.32)

RA, rheumatoid arthritis; 95% CI, 95% confidence interval.

### Annual health expenditures

The annual health expenditures were significantly larger for patients with RA than for controls. In 2013, the direct medical cost of RA patient was 2293 (± 11613, SD) US dollar/person, compared to 1199 (± 4013) US dollar/person for the control. Among the direct medical cost of RA, the cost of medications was 510 (± 2743) US dollar/person in 2013, comprising 22% of the direct medical cost. In the controls, the cost of medication was 166 (± 997) US dollar/person in 2013 (p < 0.001, compared to RA), comprising 14% of the direct medical cost. Excluding the cost of the biologic DMARDs in 2013, the cost of medications for RA decreased to 257 (± 2344) US dollar/person, comprising 11% of total direct costs.

**Fig 2:**
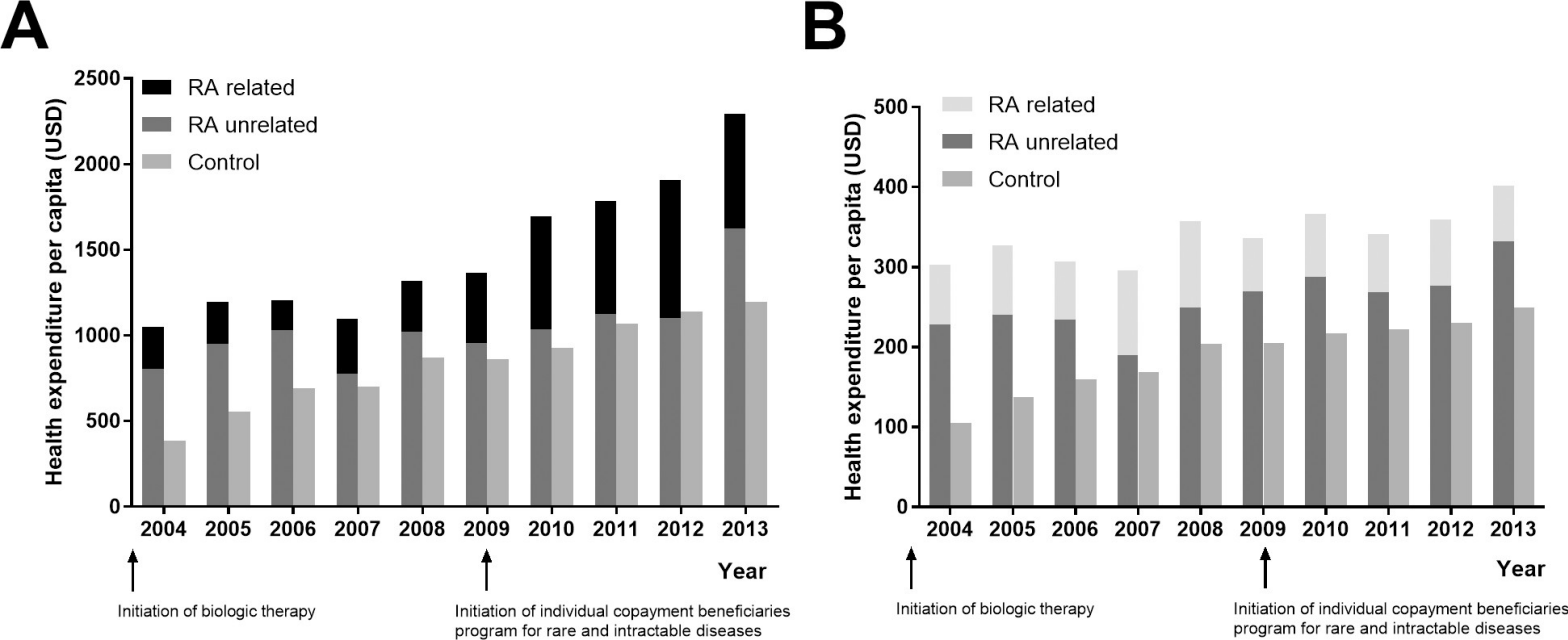
Health expenditure per capita for the rheumatoid arthritis (RA) and control group. For interpretation, 1 US dollar is equal to 1100 Korean dollars. (A) Total health care costs. (B) Amount of out-of-pocket payment.

For patients with RA, 23.91% of health costs were specifically related to the management of RA over the follow-up period. Of note, even RA-unrelated health costs were greater for the RA than control group (Fig 2A). There was a statistically significant increase in the annual health expenditures in both RA [APC 9.2 (95% CI 6.6–11.0), P < 0.05] and control groups [APC 10.0 (95% CI 7.7–12.4), P < 0.05] without any significant joinpoint (Fig 2A, S1 Fig). Although the amount of change was less stiff, the amount of out-of-pocket expenditure also increased in the RA [APC 2.6 (95% CI 0.9–4.3), P < 0.05] and control groups [APC 6.8 (95% CI 4.7–9.1), P < 0.05] without any significant joinpoint (Fig 2B, S2 Fig).

## Discussion

Biologic treatment for RA was approved in October 2003, with the first reimbursement provided in Korea in December 2003. In this study, we followed patients with an incident diagnosis of RA between January 2004 and December 2013, which is the first decade after the introduction of biologic therapies for RA. Although there was not much use in biological therapy, yet in 2013 (5.5%) it was seen that biologic treatment contributed significantly to the increase in the cost of the medication as well as the proportion of the medication costs among the direct medical costs.

Also, in July 2009, in the middle of the study period, a beneficiary program for rare and intractable diseases was initiated in Korea, with patients registered in this system, such as seropositive rheumatoid arthritis having the benefit of paying only 10% of their total medical costs, without distinction between admission and outpatient treatment. Therefore, we were able to compare the differences in the healthcare costs of patients before and after this copayment program. For the general population, the NHIS covers 80% of the total medical costs for hospitalized patients and has covered 70% for outpatient treatment until 2009. Since 2009, the proportional burden of the outpatient treatment that the patient has to bear differs among hospitals, but still covers 40–60% of the total medical cost [10].

During the follow up period, annual health expenditures increased significantly for both RA and controls. Although the initiation period of a beneficiary program for rare and intractable diseases was not showed as a significant joinpoint, the increase of the amount of out-of-pocket expenditure could be less stiff in RA group compared to controls.

The mortality rate has previously been reported to be higher among patients with RA than the general population, with a standardized mortality ratio for RA of 1.35 (95%CI 1.02–1.74) [11]. Principal cause of death was known as malignancy and cardiovascular disease, followed by respiratory disease, similar to our study cohort.

Interestingly, the mortality rate due to cardiovascular disease in our study was not higher, than that in the control group, suggesting that the risk of cardiovascular disease was not significant in the first 10 years among the incident RA patients after 2003. Similarly, a recent epidemiologic study using the Clinical Practice Research Datalink in the United Kingdom showed that death from cardiovascular disease is decreasing and there was no statistical difference in between incident RA and the control cohort since 2004 [12], and this was attributed to the contribution of the improved treatment of RA and improved management of the comorbidity. The beneficial effects of methotrexate in the treatment of cardiovascular disease would also support this contribution of better RA management to reduced cardiovascular events [13]. Among the causes of death due to the musculoskeletal disorders, there was RA itself. Because this study was based on the claims data, it was difficult to identify more detailed causes of death; however, the amyloid, therapeutic side effects, and deteriorating interstitial lung disease are likely to be relevant, and this was one of the limitations of this study.

There are other limitations to our study need to be acknowledged in the interpretation of results. First, only patients with seropositive RA were included in our study, with patients with a diagnosis of seronegative RA (ICD code, M06) being excluded. According to the other RA cohort studies in Korea [14, 15], about 85% of RA patients in Korea are expected to have rheumatoid factor of anti-CCP antibody, similar to worldwide [16, 17]. However, in the patient extraction for this study, the prevalence of seronegative RA (ICD code, M06) with prescription for more than a single DMARD was showed 1.2-fold higher prevalence than patients with seropositive RA. Therefore, there was a concern that the inclusion of seronegative RA would have biased our interpretation of results as the code used for medical expense claims would have included inflammatory diseases other than RA. Moreover, registration into the program for rare and intractable diseases, which includes seropositive, but not seronegative RA, requiring official documentation from a physician to confirm that a patient fulfills the 1987 ACR criteria. Therefore, the diagnosis of seropositive RA in Korea is conservative and reliable [7]. By excluding the seronegative RA group, the severity of RA might have been exaggerated in our cohort; however, the accuracy of the diagnosis is guaranteed.

As a result of not including cases of seronegative RA, our crude incidence rate of RA of 16.5 per 100,000 person-years is much lower than the incidence of 28.5 per 100,000 person-years reported in a recent population-based study in Korea [18] or 38.1/100,000 person-years in the United Kingdom [19].

Second, with regard to the healthcare costs, we could only analyze direct costs using the claim data from medical institutions and for medical service and medication. Although these factors are the core components of direct medical costs, there are additional direct costs, such as the use of alternative medicine, dietary supplements, and home assistance. Also, indirect costs, such as sick leaves or loss of work, are also important to consider from the perspective of the patients’ life. According to the cost of illness study conducted in Korea, between November 2009 and February 2010 [20], the mean cost for in-hospital expense and drug expense was 189,9000 KRW (about 1726 US dollars), accounting for 61% of direct costs and 29.5% of the total cost of illness. Since February 2010, direct medical costs for RA has increased, and the proportion of costs for RA drugs and the composition of indirect medical costs are likely to continue to change. Therefore, an in-depth follow-up study of the cost of illness would be needed.

Third, the maximum duration of observation in our study was only 10 years, which might be too short to evaluate the true outcomes of RA considering the chronicity of the disease. Because the treatment strategy for RA in Korea has changed after the introduction of biologic agents in 2003, long-term follow-up of patients who were prescribed with biologic agents after 2003 is warranted. From a similar standpoint, claim data do not include data after 2013. The rate of use of biologic agents was only 6% in our current dataset, which is similar to that reported in a previous Korean cohort study conducted in 2012 [15] and a study in Western Europe in 2011 [21]. However, the rate of use of biologics would be expected to be higher in 2018. Thus, the status of Korean patients with RA presented in our study may not be up-do-date and a follow-up study would be needed. Therefore, we expect the prospective national-wide multicenter cohort for longer period to overcome these limitations.

To our knowledge, this is the first study to have quantitatively assessed the status of mortality, disability, and healthcare costs among Korean patients with RA by using representative claims data of the NHIS, which covers most of the medical usage of the Korean population. Because we used a balanced sample cohort, our findings can be considered as being representative of the entire Korean population of patients with and without RA.

Our findings indicate that RA is not only a painful disease of the musculoskeletal system, but also carries a high risk of high mortality and disability, as well as being associated with high healthcare costs. The higher healthcare costs for patients with RA, compared to the control group, were associated to the direct costs of RA and to health comorbidities. Although the rate of use of biologic agents was only 6% in our study cohort, the annual health costs increased significantly over the 10-year period of observation, and this in both the RA and control groups. It is noteworthy that although the start of the insurance benefit program did not influence the joinpoint, the increase in the medical expenses for the RA group was largely buffered by the health insurance system due to the increase in the proportion of coinsurance. Further research regarding the mortality trends of RA would be needed to see whether this increase in the cost of health care and social support will result in the reduction or RA mortality. Further research is also needed to identify the determinants of disability and mortality among patient with RA.

Of note, as infection and pneumonia were major contributing factors to the higher mortality rate among patients with RA, early diagnosis and active treatment of infectious disease is warranted. Preventive measures such as influenza or pneumococcal vaccination might also be useful to reduce the burden of disease for patients, the healthcare system and society. Also, as the cost of medical care for patients with RA continues to increase, there is a need to consider who, society or patients, should bear the economic burden of the disease.

## Supporting information

S1 Fig Joinpoint graph of the temporal trend in annual health expenditures between the incident rheumatoid arthritis (RA) and control groups. (DOCX)

S2 Fig Joinpoint graph of the temporal trend in the mortality rate between the incident RA and control groups. (DOCX)

S1 Text The RECORD statement–checklist of items, extended from the STROBE statement, that should be reported in observational studies using routinely collected health data. (DOCX)

S2 Text Extended guidelines: Definition of physical disability due to joint disorders, according to the National Health Insurance Service in Korea. (DOCX)
