# Supplementary material for: Mortality, disability, and healthcare expenditure of patients with seropositive rheumatoid arthritis in Korea: A nationwide population-based study
Source: PLoS One. 2019 Jan 8;14(1):e0210471. doi: 10.1371/journal.pone.0210471 (PMC6324802; doi:10.1371/journal.pone.0210471)
Supplement: S1 Fig — (DOCX) [file pone.0210471.s001.docx]

S1 Fig. Joinpoint graph of the temporal trend in annual health expenditures between the incident rheumatoid arthritis (RA) and control groups.


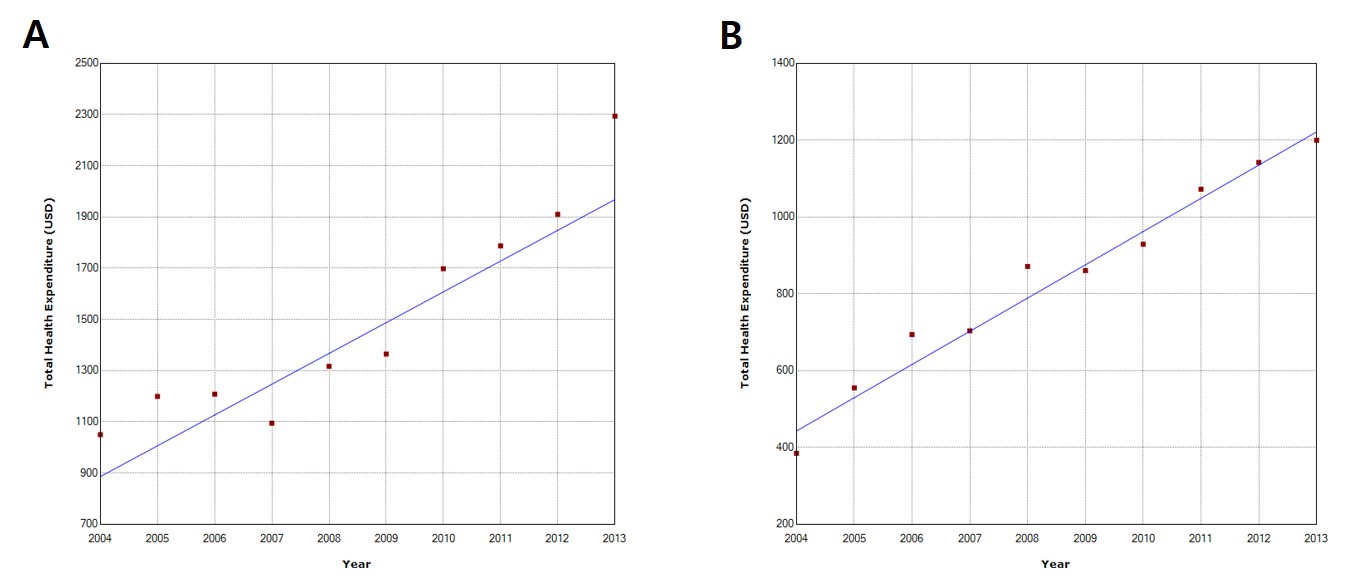


Joinpoint graph (A) shows an increase in the annual health expenditures in the incident RA group over the calendar year subsequent to the diagnosis, without a significant joinpoint. Joinpoint graph (B) shows an increase in the annual health expenditures between the incident RA and control groups, matched for sex, age, follow-up duration, geographic region, and household income for consecutive calendar years, without a significant joinpoint.
