# Supplementary material for: Mortality, disability, and healthcare expenditure of patients with seropositive rheumatoid arthritis in Korea: A nationwide population-based study
Source: PLoS One. 2019 Jan 8;14(1):e0210471. doi: 10.1371/journal.pone.0210471 (PMC6324802; doi:10.1371/journal.pone.0210471)
Supplement: S2 Fig — (DOCX) [file pone.0210471.s002.docx]

S2 Fig. Joinpoint graph of the temporal trend in the mortality rate between the incident RA and control groups.


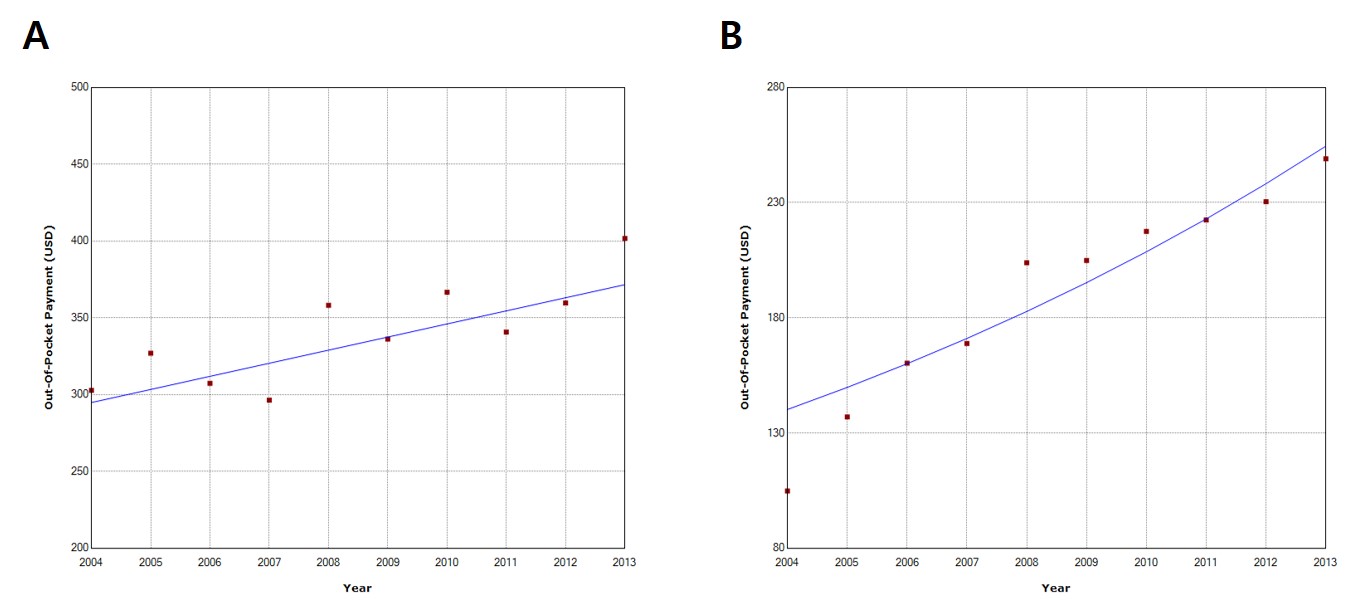


Joinpoint graph (A) shows an increase in the out-of-pocket payment in the incident RA group over the calendar year subsequent to the diagnosis, without a significant joinpoint. Joinpoint graph (B) shows an increase in the out-of-pocket payment between the incident RA and control groups, matched for sex, age, follow-up duration, geographic region, and household income for consecutive calendar years, without a significant joinpoint.
