# Supplementary material for: Mortality, disability, and healthcare expenditure of patients with seropositive rheumatoid arthritis in Korea: A nationwide population-based study
Source: PLoS One. 2019 Jan 8;14(1):e0210471. doi: 10.1371/journal.pone.0210471 (PMC6324802; doi:10.1371/journal.pone.0210471)
Supplement: S2 Text — (DOCX) [file pone.0210471.s004.docx]

**S2 Text.** Extended guidelines: Definition of physical disability due to joint disorders, according to the National Health Insurance Service in Korea

**A. Overview**

A1. Joint disability refers to joint stiffness, muscle weakness, or joint instability (either for the native joint or joint arthroplasty).

A2. Joint stiffness includes ankylosis (with the joint fixed in one position, complete stiffness) and reduced range of motion (partial stiffness). The extent of joint range of motion is measured using a goniometer, and compared to the normative range of joint motion (%).

A3. At this time, the range of motion of the joint is based on the passive range of motion. The measure is obtained after repeated passive joint motion over several minutes, with the examiner applying a force of 0.5 kg.

However, in cases of muscle paralysis, tendon rupture, or traumatic muscle rupture, where the range of active joint motion is significantly smaller than that of the passive range of motion, although there is a functional disability, the diagnosis of ‘joint disorder’ is applied as per the guidelines, using the active rather than passive range of motion.

A4. When further investigations apart from the physical examination are necessary for diagnosis, radiological examination and/or electromyography is recommended.

B. Joint disability of the upper limb

| Disability grade | Description |
| --- | --- |
| Grade 1 Class 1 | - Range of motion of the shoulder, elbow, and wrist, bilaterally, is >75% |
| Grade 2 Class 1 | - Reduction in the range of motion of the shoulder, elbow, and wrist, bilaterally, of >75% |
| Grade 2 Class 2 | - Reduction in the range of motion of >75% in 2 of the 3 major joints of the upper limb.  - Reduction of 50% to 75% across all 3 major joints of the upper limb. |
| Grade 2 Class 3 | - Reduction of >75% of the total range of motion of the digits of the hand. |
| Grade 3 Class 1 | - Reduction of >50% but <75% in the range of motion of the 3 major joints of the upper limb.  - Reduction of >25% but <50% in the range of motion of the 3 major joints of the upper limb. |
| Grade 3 Class 2 | - The total range of motion of the joints of the thumb and the second finger, bilaterally, is reduced by >75%. |
| Grade 3 Class 3 | - The total range of motion of all fingers in one hand is reduced by >75%. |
| Grade 3 Class 4 | - Reduction of >75% in the range of motion of the 3 major joints of the upper limb.  - Reduction of >50% but <75% in the range of motion of the 3 major joint of the upper limb. |
| Grade 4 Class 1 | - A reduction of >75% at 1 of the 3 major joints of the upper limb (shoulder, elbow or wrist), in one arm.  - A reduction of >75% in the range of motion of the thumb, bilaterally. |
| Grade 4 Class 2 | - Reduction of >75% of the total range of motion of the joints of the thumb and second finger of one hand. |
| Grade 4 Class 3 | - The total range of motion of three fingers, including the thumb or second finger, of one hand is reduced by >75% |
| Grade 4 Class 4 | - The total range of motion of four fingers, including the thumb or second finger, of one hand is reduced by >50% and <75%. |
| Grade 5 Class 1 | - Reduction of >50% and <75% of the 3 major joints of one upper limb.  - Reduction of >25% and <50%. The range of motion of all three joints in one arm is reduced by more than 25% and less than 50% |
| Grade 5 Class 2 | - The total range of motion of the joints of the thumbs of both hands is reduced by more than 50% and less than 75% |
| Grade 5 Class 3 | - The total range of motion of the joints of the thumbs of one hand has been reduced by more than 75% |
| Grade 5 Class 4 | - The total range of motion of the joints of the thumb and the second finger of one hand is reduced by more than 50% and less than 75%, respectively. |
| Grade 5 Class 5 | - The total range of motion of the three fingers including the thumb or second finger of one hand is reduced by more than 50% and less than 75%, respectively. |
| Grade 6 Class 1 | - Reduction of >50% of the range of motion of 1 of the 3 major joints in one upper limb (shoulder, elbow, or wrist joint).  - Reduction of >50% and <75% of the range of motion of the thumb of one hand. |
| Grade 6 Class 2 | - Reduction of >75% in the range of motion of two fingers, including the second finger, of one hand. |
| Grade 6 Class 3 | - Reduction of >50% and <75% in the total range of motion of the joints of two fingers, including the thumb, of one hand. |
| Grade 6 Class 4 | - Reduction of >75% of the total range of motion of the joints of the third, fourth and fifth fingers of one hand. |

※ The three major arms of the upper limb are the shoulder, elbow, and wrist.

※ The three joints of the fingers are the carpal-metacarpal joint and the proximal and distal inter-phalangeal joint.

※ In cases of prosthetic arthroplasty of the shoulder, elbow, and wrist, when the prognosis is poor (including obvious osteolysis, loosening of the implant, unstable instability, and inflammation), disability is quantified as ‘Class 1’ (involving 2 or 3 joints) or grade 6 ‘Class 2’ (involving 1 joint). Of note, this classification does not apply to patients who have undergone partial arthroplasty, such as arthrodesis of the elbow joint and distal ulna arthroplasty of the wrist joint; therefore, the extent of disability in these cases is not recognized.

- Moderate instability refers to the occurrence of radiographic subluxation or a reduction in joint angular range of motion of >50%.

C. Joint disability of the lower limb

| Disability grade | Description |
| --- | --- |
| Grade 1 Class 2 | - Reduction of >75% of the total range of motion of all 3 major joints of the lower limb, bilaterally. |
| Grade 2 Class 4 | - Reduction of >50% and <75% of the total range of motion of all 3 major joints of one lower limb.  - Reduction of >50% and <75% of the total range of motion of all 3 major joints of the lower limb, bilaterally. |
| Grade 3 Class 5 | - Reduction of >75% of the total range of motion of all 3 major joints on one lower limb. |
| Grade 4 Class 1 | - Reduction of >50% and <75% of the total range of motion of all 3 major joints of the lower limb, bilaterally.  - Reduction of >25 and <50% of the total range of motion of all 3 major joints of the lower limb, bilaterally. |
| Grade 4 Class 2 | - Reduction ≥90% of the total range of motion of the ankle and knee of one lower limb. |
| Grade 4 Class 5 | - Reduction of >75% of the total range of motion of all 3 joints of one lower limb, with a reduction >75% in functional weight-bearing activity  - Reduction of >50% and <75% of the total range of motion of all 3 major joints of one lower limb. |
| Grade 5 Class 1 | - Reduction of >75% of the total range of motion of the hip or knee in one lower limb. |
| Grade 5 Class 2 | - Reduction of ≥90% in the range of motion of the ankle joint (or complete stiffness) in one lower limb. |
| Grade 5 Class 6 | - Reduction >50% and <75% in the range of motion of the 3 major joints of one lower limb.  - Reduction of >25% and <50% in the range of motion of all 3 major joints of one lower limb. |
| Grade 5 Class 7 | - Reduction of >75% in the total range of motion of all toes of both feet. |
| Grade 6 Class 2 | - Reduction of >50% in the range of motion of the hip or knee joint of one lower limb. |
| Grade 6 Class 3 | - Reduction of >75% of the range of motion of the ankle joint of one lower limb. |

※ The three major joints of the lower limb are the hip, knee, and ankle.

※ In cases of prosthetic arthroplasty of the hip, knee, and ankle, when the prognosis is poor (including obvious osteolysis, loosening of the implant, unstable instability, and inflammation), disability is quantified as ‘Class 1’ (involving 2 or 3 joints) or grade 6 ‘Class 2’ (involving 1 joint). Of note, this classification does not apply to patients who have undergone partial arthroplasty, such as patellar resurfacing; therefore, the extent of disability in these cases is not recognized.

- Moderate instability refers to the occurrence of radiographic subluxation or a reduction in the joint angular range of motion of >50% or a specific reduction in the range of motion of >75% at the ankle.

※ A joint disorder involving the hip and/or knee is quantified as a grade 6 class 2 joint disorder.

A) Use of a brace due to a joint position 10 mm anterior or posterior to the normative position, by objective measure.

   B) Severe limitation in activities of daily living because of habitual dislocation.
